# The Contribution of Epigenetic Inheritance Processes on Age-Related Cognitive Decline and Alzheimer’s Disease

**DOI:** 10.3390/epigenomes5020015

**Published:** 2021-06-18

**Authors:** Aina Bellver-Sanchis, Mercè Pallàs, Christian Griñán-Ferré

**Affiliations:** Pharmacology Section, Department of Pharmacology, Toxicology, and Therapeutic Chemistry, Faculty of Pharmacy and Food Sciences, Institute of Neuroscience, University of Barcelona (NeuroUB), Av Joan XXIII 27-31, 08028 Barcelona, Spain; abellversanchis@gmail.com (A.B.-S.); pallas@ub.edu (M.P.)

**Keywords:** epigenetic mechanisms, learning process, memory formation, cognitive decline, intergenerational epigenetic inheritance, transgenerational epigenetic inheritance, AD

## Abstract

During the last years, epigenetic processes have emerged as important factors for many neurodegenerative diseases, such as Alzheimer’s disease (AD). These complex diseases seem to have a heritable component; however, genome-wide association studies failed to identify the genetic loci involved in the etiology. So, how can these changes be transmitted from one generation to the next? Answering this question would allow us to understand how the environment can affect human populations for multiple generations and explain the high prevalence of neurodegenerative diseases, such as AD. This review pays particular attention to the relationship among epigenetics, cognition, and neurodegeneration across generations, deepening the understanding of the relevance of heritability in neurodegenerative diseases. We highlight some recent examples of EI induced by experiences, focusing on their contribution of processes in learning and memory to point out new targets for therapeutic interventions. Here, we first describe the prominent role of epigenetic factors in memory processing. Then, we briefly discuss aspects of EI. Additionally, we summarize evidence of how epigenetic marks inherited by experience and/or environmental stimuli contribute to cognitive status offspring since better knowledge of EI can provide clues in the appearance and development of age-related cognitive decline and AD.

## 1. Introduction

Observations of inheritance of the genomic expression state not following Mendelian laws drew scientists’ attention many years ago. Abundant evidence shows that the environment can reversibly modulate the gene expression and, thus, fits well with the idea that the genome is not static as once thought. In 1942, Waddington coined the term “epigenetics” for the first time to describe the bridge between genotype and phenotype during development [1]. Albeit the term has been redefined multiple times, we refer to epigenetics as the mechanisms that maintain the memory of a phenotype chromosome without alterations in the DNA sequence, serving as an important bridge between environmental stimuli and gene expression. These molecular events can occur in the form of chromatin remodeling, covalent modifications of DNA, post-translational modifications (PTMs) histones, and activity of non-coding RNAs (ncRNA), among others [2,3]. Furthermore, epigenetic mechanisms can modulate from transgenerational inheritance to gene activity maintenance throughout life such as adult neurons [4].

Several studies accept that changes in epigenetic modifications are associated with aging [2,4,5]. Aging is an inevitable outcome of life characterized by the progressive functional decline of organisms at the molecular, cellular, and/or physiological levels. This biological process is one of the main factors for human diseases such as cancer, neurodegeneration, and cardiovascular diseases [6]. Similarly, it has been described that epigenetic mechanisms play an important role in the development of neurodegeneration [2]. These mechanisms have been involved in the functioning of the nervous system, being responsible for early developmental programming [7,8], responses to external or environmental stimuli [9,10,11], and participating in neurological disorders and in cognition, activity-dependent changes in synaptic plasticity [12,13], and learning and memory formation [9,14,15,16,17].

Memory is the ability to acquire, store, and retrieve learned information and is crucial for individual adaptive behavior [18]. There are two major types of memories: short-term memories, which last for a few hours, and long-term memories, which persist for several days or longer. Understanding the underlying basis of how memory remains is still a pending task in the field of neuroepigenetics. Richard Semon first coined “the memory engram” [19], suggesting that learning induces persistent changes in specific brain cells that retain information and are subsequently reactivated under the proper conditions of recovery [20]. Moreover, Roberson and Sweatt described the “mnemogenic reactions”, which are a chain of biological reactions that occurs to store long-term memory after its formation. Examples of these mechanisms are de novo protein synthesis and DNA histone modifications [21]. Therefore, epigenetic modulation has been associated with learning and memory, and many recent studies have shown that these modifications could support memory formation and maintenance through a cascade of specific changes to gene expression, including enduring memories. Likewise, those epigenetic modifications play an important role in the function and homeostasis of the central nervous system (CNS). Indeed, a growing body of evidence reported that the CNS’s regulation associated with long-term changes in gene transcription is mediated by modulation of chromatin structure. This regulation is critical because approximately 80–95% of protein-coding genes are expressed in the human brain throughout the organisms’ lives, so a further understanding of CNS’s regulation is a really big necessity [22]. Therefore, the epigenetic marks accumulated and maintained within the epigenome throughout life carry important information about the interaction between the individual and their environment. Most human diseases, including neurodegenerative disorders, result from alterations in multiple molecular pathways together with the interaction of an environmental factor [3]. For instance, it has been described that Alzheimer’s disease (AD) might have at least a partial epigenetic etiology [2,16,23].

Here, we review the contribution of epigenetic inheritance (EI) in age-related cognitive decline, which plays an important role in our understanding of disease and disease risk. We first describe the prominent role of epigenetic factors in memory processing and its contribution to neurodegenerative diseases. Then, we discuss aspects of EI, briefly describing the mechanisms involved. Further, we summarize evidence of how epigenetic marks inherited by experience and/or environmental stimuli contribute to cognitive status offspring, focusing on the relationship among epigenetics, cognition, and neurodegeneration disorders, such as AD. On the one hand, this information can be very relevant for the offspring, providing an adaptive advantage [24,25]. On the other hand, the accumulated epigenetic load could also trigger risk factors for cognitive disorders [26]. Thus, we conclude with some future perspectives, since the target of modulation to a new environment and/or stimulus could help better prepare the offspring for the challenging environmental conditions they might encounter during their lives, showing the great advantage of EI over the classical inheritance.

## 2. The Role of Epigenetic Mechanisms in Learning and Memory Formation

As we aforementioned, memory formation allows us to acquire information and store it, producing long-lasting brain and behavior changes [27]. Learning and memory formation requires the structure and functional remodeling of synapses through regulated cellular and molecular machines. Neuronal activation triggers the modification, trafficking, and synthesis of new proteins from memory-related molecules through intracellular signaling pathways, gene transcription, and protein synthesis [28]. However, understanding the underlying mechanisms of how these changes in memory-related molecules are maintained for the long-term in supporting various cellular events during memory formation, consolidation, and retrieval has emerged as the main goal in the neuroscience field. Many studies have shown evidence of the changes in active epigenetic markers during learning and memory processes [29,30]. DNA is packaged into chromatin within the nucleus. Overall, this supranucleoprotein complex is composed of DNA, histones, non-histone proteins, and interacting RNA molecules [31]. Among other processes, which occurs at different levels, gene expression regulation is especially crucial for proper memory processing, as some genes need to be activated while some genes must be suppressed [31]. Indeed, chromatin may adopt one of two major states interchangeably, between heterochromatin (a compact form) and euchromatin (a relaxed form) states. In other words, heterochromatin is resistant to the binding of various proteins, such as transcriptional machinery, whereas euchromatin is open to modifications and transcriptional processes [32].

**Figure 1:**
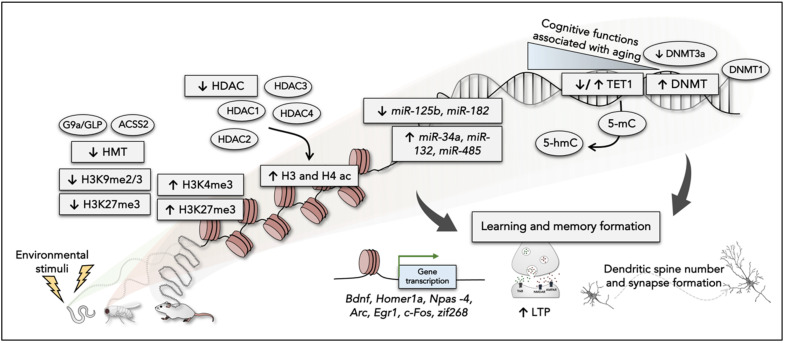
Overview of the findings of epigenetic alterations promoting learning and memory formation.

A body of evidence clearly showed the interplay between the process of learning and memory and the structural changes in chromatin associated with gene regulation (Figure 1). Several studies supported the critical role of DNA methylation in memory formation and maintenance and neuronal function, particularly in some brain regions, such as the hippocampus, prefrontal cortex, and amygdala [33]. Approximately 60–80% of the CpG dinucleotides in the human genome are methylated, mainly catalyzed by DNA methyltransferases (DNMTs). Genetic and/or pharmacological inhibition of DNMTs impaired memory consolidation in various behavioral tasks in various brain regions [9,34,35,36,37,38,39,40]. Likewise, recent work observed increased spatial memory and learning-induced activity of ten-eleven translocation 1 (TET1), a methylcytosine dioxygenase that catalyzes the oxidation of 5-methylcytosine (5-mC) to 5-hydroxymethylcytosine (5-hmC) [41,42]. However, in the opposite direction, a work reported that Tet1 knock-out (KO) downregulated expression of multiple neuronal activity-regulated genes, including Neuronal PAS Domain Protein 4 (*Npas4*), *c-Fos*, and Activity Regulated Cytoskeleton Associated Protein (*Arc*), and impaired Morris water maze (MWM) memory [43].

Moreover, DNA methylation inhibits the binding of transcriptional machinery inducing gene silencing [33]. In this regard, Miller et al. [36] found that the synaptic plasticity gene reelin was demethylated and transcribed. However, while it is true that DNA methylation is typically related to the repressions of gene transcription inhibiting the binding of transcriptional machinery to binding sites, another study suggests that DNA methylation repress the expression of memory suppressor genes, such as protein phosphatase 1 (PP1), and thus may regulated memory interacting with histone acetylation levels [44]. These findings illustrate that various DNA methylation forms respond to learning and are involved in the memory consolidation process. Furthermore, DNA methylation is dynamically regulated since memory formation requires hypermethylation of memory suppressor genes and hypomethylation of promoter genes.

On the other hand, a wealth of studies revealed that chromatin modifications are involved during memory formation and are related to gene expression. Patterns of histone PTMs were important for memory consolidation and retention in mice that decreased PP1 levels [45]. Regarding its role, many groups have since demonstrated that histone deacetylases (HDACs) activity and/or inhibition of histone acetyltransferase (HAT) activity promote the reductions of histone acetylation patterns, impairing memory [46,47,48,49,50,51,52], enhancing long-term potentiation (LTP) [53,54,55], and increasing synaptic plasticity [56,57,58,59]. An early study showed that gene expression and epigenetic alterations are required for long-term memory-related synaptic plasticity in *Aplysia* sensory neurons. It has been described that histone acetylation was caused by the facilitatory transmitter 5-HT activating cyclic AMP-responsive element-binding (CREB) protein 1 (CREB1). In contrast, histone deacetylation was related to the inhibitory transmitter FMRFa, which causes CREB2 activation [56]. Likewise, histone phosphorylation has been begun to be highlighted in initial memory formation [15]. Nevertheless, unlike histone acetylation or phosphorylation are often associated with transcriptional activation [50], histone methylation can modulate transcriptional activation and repression [47]. Although histone methylation appears to have opposites functions, Gupta and colleagues [15] suggest that active gene expression and repression are necessary for memory formation. Transcription of memory supporting genes was associated with the increase of histone H3 lysine 4 trimethylated (H3K4me3) [51], whereas its inhibition was mediated by histone H3 lysine 9 dimethylated (H3K9me2) mark [15,51]. Regarding this last epigenetic mark, inhibition of G9a/GLP, a histone methyltransferase (HTM), enhanced long-term memory formation and was accompanied by increased acetylation of H3K9 in the entorhinal cortex [60]. Moreover, alterations of the activity of histone-modifying enzymes, including CREB binding-protein, have been described to affect memory storage [61].

Additionally, last but not least, the emerging interest of active microRNAs (miRNAs) highlighted its role in mediating the regulation of gene transcription in the initial formation or fear extinction memory [62,63,64,65] (see Table 1 in [66]). Specific miRNAs involved in the regulation of dendritic arborization and synaptogenesis. Among them, miR-125b negatively regulate synaptic plasticity via targeting NR2A mRNA, *miR-132* overexpression modulates synapse number and miniature excitatory postsynaptic currents (mEPSC) [67]. Moreover, *miR-485* colocalized with synaptic vesicle glycoprotein (SV2A) in dendrites and was shown to regulate dendritic spine number and synapse formation [68]. Besides, *miR-34a* and *miR-182* were actively regulated in the amygdala when fear memory formation [63,64].

## 3. Epigenetic Deregulation in Neurodegenerative Diseases: AD as a Model

Globally, 50 million people are affected by dementia, increasing to 152 million in 2050 [69]. Hence, the prevalence of AD in people of 65 years of age and older increases by a factor of two every five years [70,71]. The most common neurodegenerative disease is AD, which is associated with progressive and irreversible neurodegeneration. Clinically, AD is characterized by behavioral, functional, and cognitive deficiencies; and molecularly, the deposition of extracellular amyloid-β (Aβ) plaques and the aggregation of intracellular neurofibrillary tangles (NFTs) containing hyperphosphorylated Tau protein [72,73] are two of the pathology’s hallmarks.

Given that the main risk factor for AD is aging, which is associated with cognitive decline, chromatin alteration that occurs in the old brain might therefore be crucial targets to prevent cognitive deterioration [74]. In this regard, numerous studies showed growing evidence that epigenetic dysregulations are involved in AD. Thus, scientists suggest that epigenetic mechanisms play an important role in the development of neurodegeneration. These mechanisms have been involved in the functioning of the nervous system and participate in neurological disorders and in cognition, learning, and memory formation. The following table (Table 1) compiles some of the findings where epigenetic mechanisms modulated critical events in AD.

**Table 1:** Alteration of epigenetic mechanisms observed in AD.

Epigenetic Mechanism	Epigenetic Alteration	Levels in AD	Model	Outcome	Refs
DNA methylation	*Dnmt1* *Dnmt3a*	↓	DKO mice	Loss of LTP at CA1 synapses in the hippocampus and deficits in hippocampus-based learning and memory	[34]
	*Tet1*	↓	C57B6/L mice	Deficit in long-term contextual fear memory	[41]
	*TREM2*	↑	Human	Increased immune genes	[75]
	*PIN1*	↓	Human	Increased AD risk	[76]
	*TNF*-*α*	↓	Human	Encodes multifunctional pro-inflammatory cytokines	[77]
	*GSK3B*	↑	Human	Increased Aβ deposition and NFTs	[78]
	*IL-6* *IL-1β*	↑	Human	Increased inflammatory responses	[79]
	APP *	↓	Human	Increased Aβ deposition	[78,80,81]
	*MAPT*	↑	Human	Increased Tau protein levels	[82]
	*PSEN1*	↓	TgCRND8 mice Human	Increased Aβ deposition	[83,84]
Histone modifications	HDAC1 HDAC2	↑	HDACKO mice Ck-p25 mice Sprague-Dawley rats	Increased Aβ deposition Block expression neuroplasticity genes Reduces the histone acetylation of important genes for learning and memory Decrease of dendritic spine density, synapse number	[47,57,85]
	HDAC3	↑	HDAC3-Flox mice	Impairment of long-term memory for object recognition	[55]
	HDAC4	↓	HDAC4KO mice	Impairment of synaptic plasticity and memory formation	[59]
	HDAC6	↑	HDAC6KO mice	Potential modulator of Tau phosphorylation and its aggregations	[86]
	SIRT1	↓	N2aSwe/APP cells SIRT-null and SIRT1^F/F^ mice Human	Increased formation of Aβ peptides Downregulation of alpha-secretase ADAM10 Tau protein aggregation	[87,88]
	H3K9ac	↓	Long-Evans rats	Impairment of learning process	[89]
	H3K27ac	↑↓	Ck-p25 mice C57BL/6mice Human	Increased immune genes Decreased on synaptic plasticity genes	[90,91]
	H4K12ac	↓	C57BL/6mice	Age-related memory loss	[92]
	H3K4me3	↑	Fischer-344 rats C57BL/6mice	Increased of somatostatin and cortistatin genes Age-related memory decline	[93,94]
	H3K9me2	↑	Fischer-344 rats	Decreased *Bdnf* transcription Age-related memory decline	[93]
	H3K36me	↓		Age-related memory decline	[95]
	H3K79me		SAMP8		
	H4K20me				
miRNA	*miR-29*	↓	Human	Increased *Bace1* expression Increased Aβ deposition	[96]
	*miR-107*				
	*miR-132*	↓	APP/PS1 mice	Increased Aβ deposition Increased Tau hyperphosphorylation	[97]
	*miR-138*	↑	N2a/APP and HEK293/Tau cells	Increased Tau hyperphosphorylation	[98]
	*miR-195*		Sprague-Dawley rats		[99]
	*miR-206*	↑	Tg2576AD mice Human	Downregulation of BDNF gene expression	[100]
	*miR-132*	↓	miR-132/212 KO mice Human	Tau protein overexpression, hyperphosphorylation, and aggregation	[101]
	*miR-219*	↓	*D. melanogaster* that produces human Tau	Block of repression Tau synthesis	[102]

Abbreviations: DKO: double knock-out; *TREM2*: triggering receptor expressed on myeloid cells 2; *PIN1*: peptidyl-prolyl cis-trans isomerase NIMA-interacting 1; *TNF-α*: tumor necrosis factor-α; *GSK3B*: glycogen synthase kinase 3 beta; *IL-6*: interleukin 6; *IL-1β*: interleukin 1β; *APP*: amyloid-beta precursor protein; *MAPT*: microtubule-associated protein tau; SIRT1: sirtuin 1; *Bace1*: β-secretase-1; ADAM10: A disintegrin and metalloproteinase 10; *Bdnf*: brain-derived neurotrophic factor; *D. melanogaster*: *Drosophila melanogaster*. * These studies used a small cohort, and the findings could not be confirmed in a larger cohort [103].

## 4. Epigenetic Inheritance (EI)

In general, epigenetic changes that occur during the lifetime of an individual’s germline are not generally thought to be inherited into subsequent generations. Indeed, DNA and histone modifications are erased and re-established in each generation through a developmental reprogramming process by modifications by various epigenetic modifying enzymes, histone variant replacement, and chromatin remodeling enzymes [104,105]. Thus, in each reprogramming window, a specific set of mechanisms regulates the erasure and re-establishment of epigenetic changes. Epigenetic reprogramming occurs during development at two distinct stages: in primordial germ cells (PGCs), once they have reached the embryonic gonads, and in the early embryo beginning in the zygote [106]. Regarding the role of DNA methylation during reprogramming, there are at least three rounds. The first occurs just after fertilization, in the zygote and early cleavage stages, to erase gametic epigenomic marks.

Moreover, the other reprogramming process occurs in the germline, where the paternal and maternal somatic programs are erased [105]. Besides, the last wave of epigenetic reprogramming occurs in the developing germline; the post-migratory PCGs residing in the genital ridges undergo genome-wide DNA demethylation, which includes erasure of genomic imprints and extensive chromatin remodeling [107]. In addition, a global loss of several histone modifications, such as H3K27me3 and H3K9ac, is observed after demethylation of DNA, indicating that widespread DNA repair might also be associated with global remodeling of nucleosomes in PGCs [108]. After all, the epigenome reaches its most ‘naive’ state during development and sets the scene for the acquisition of new epigenetic information and genomic imprints. However, there is evidence that at least some epigenetic alterations can escape this re-establishment of the epigenome [109], becoming evident that the environment experienced during an individual’s lifetime might impact their health and influences the vulnerability of offspring to many pathological conditions [110].

Therefore, in some cases, these effects may be transmitted for several generations, even if the environment has reverted. EI is defined as the transmission of non-DNA base sequence information between generations via the germline [111,112]. Exposure in the parenteral generation (P0) to environmental stressors can affect offspring health increasing the risk for specific phenotypes in subsequent generations (first filial generation (F1), second filial generation (F2), third filial generation (F3), and next generations. When the exposure is maternal, the F1 is directly exposed to the environment as fetuses in utero. Besides, its PGCs, which will become the F2, are also subjected to the stressor. Due to them already present and developing in the uterus.

**Figure 2:**
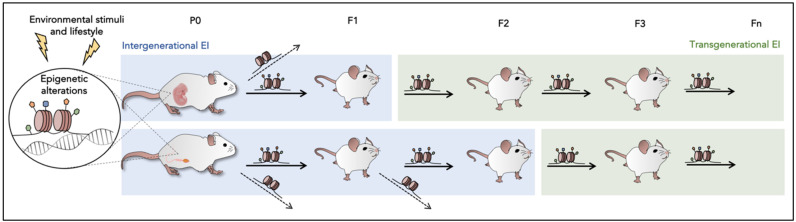
Comparison of epigenetic inheritance between the paternal and maternal lineages (the mouse model has been chosen as an example).

In contrast, P0 and his PGCs that give rise to the F1 generation are directly exposed to the environmental insult in the paternal linage. Intergenerational EI refers only to the generations that were directly exposed to the environment. Thus, it comprises the transmission of epigenetic marks from one generation to the next, up to F1 in paternal lineage, while in maternal lineage, it would be considered up to F2. This includes parental–effect phenotypes, such as the passage of RNA molecules and maternal proteins from oocytes to embryos, or it can be mediated by chromatin changes in the exposed fetuses or germ cells that are not inherited further. Otherwise, the passage of information in the F2 in the case of paternal transmission or F3 in the case of maternal transmission, which was never exposed to parental stress, is defined as a transgenerational epigenetic inheritance (TEI) [113,114] (Figure 2). TEI supports stable epigenetic changes that persist through epigenetic reprograming and are transmitted to the newly formed germ cells. In the case of chromatin marks that involve histones or DNA methylation, the transgenerational effects are generally short-lived, lasting three or four generations [115,116]. Small RNA-mediated inheritance is also short-lived, although it is heritable for up to 80 generations in *Caenorhabditis elegans* (*C. elegans*) [117].

## 5. Brief Understanding of Mechanism Candidates in EI

The mechanism underlying EI is currently at the forefront of epigenetic research using worms, flies, and rodent models. At this point in our review, we briefly describe the molecular understanding of the epigenetic changes that underlie phenotypes and how they are transmitted and maintained for subsequent generations. These molecular mechanisms of inheritance might contribute to epigenetic memory on their own or in different combinations.

Notably, DNA methylation is considered an important mechanistic candidate for EI, being its role often discussed. To consider DNA methylation as a heritable epigenetic mark, it should be environmentally modulated, mitotically or meiotically stable [111,112], escaping the epigenetic reprogramming PGCs and post-fertilization embryos [118,119]. In fact, this epigenetic “erasure” generates a totipotent state required to form subsequent generations, resetting epigenetic marks [120]. Thus, abnormal DNA methylation patterns caused by environmental stressors would have to generate resistance to reprogramming to appear and cause phenotypes to the next generations. Interestingly, 5-mC within specific genomic regions, including repeat sequences, such as intracisternal A particles (IAPs), and rare regulatory elements is resistant to resetting, maintaining genomic stability during widespread erasure [121], for example, 5mC has often been proposed as a mechanism underlying TEI [122]. One of the most classic TEI models involving an IAP element is the agouti viable yellow (A^vy^) epiallele [109]. It is well described that hypomethylation of a cryptic promoter in the IAP element upstream of the agouti gene is inherited over several generations through the maternal line [109,123] and can be manipulated by environmental factors [124,125]. Beyond A^vy^ ’s model, it has not been easy to identify differentially methylated regions (DMR) in the genome that were stable for multiple generations correlated with a phenotype.

Additionally, similar resetting events also occur during histone modifications, although the role of histone alterations remain unclear. In many organisms, most of the histones in sperm are globally removed during spermatogenesis and replaced by protamines, allowing for supercompaction of DNA [33]. Furthermore, retaining some histone modifications in the germline and zygote during epigenetic re-establishment forms another possibility of inheritance to subsequent generations, while histones are retained throughout the genome in the oocyte [126,127,128]. It has recently been suggested that histone alterations and their regulatory enzymes involve epigenetic memory across generations. For example, a polycomb repressive complex 2 (PRC2) transmits memory of X-chromosome repression transgenerationally regulating H3K27me3 in *C. elegans* [129]. Likewise, in the mouse model, the ectopic expression of lysine-specific histone demethylase 1A (KDM1a), a human H3K4 demethylase, during spermatogenesis causes developmental abnormalities up to F3 [130]. Nevertheless, wild-type (WT) sperm of the F1 generation presented standard H3K4me2 profile and standard DNA methylation patterns. Therefore, while it is true that disruption of the histone methylation machinery may initiate TEI, a second epigenetic mechanism may be necessary.

Remarkably, ncRNAs have been well-studied as a mechanistic candidate of TEI research. While the manner in which environmental stressors experienced by adult somatic cells affect the establishment or maintenance of methylation of DNA and histone modifications in gametes remains unclear, extensive evidence that transmission epigenetic information from somatic to germline RNA has helped clarify our understanding of epigenetic inheritance. Small ncRNAs act as sequence guides directing DNA or histone methylation and by post-transcriptionally regulating mRNA [131]. One of the best models to study RNA inheritance was *C. elegans* [117]. Starvation-induced expression of small RNAs or exogenous RNA interference (RNAi) resulted in heritable gene silencing that persists for multiple generations [132,133].

Moreover, it was hypothesized that piwi-interacting RNA (piRNA), which typically mediated transposon silencing in the germline, and exogenous RNAi might converge into a common pathway requiring small secondary RNAs and chromatin regulatory complexes to ultimately bring about stable TEI [132]. Otherwise, to cause phenotypes in mice, the ncRNA sperm exposed to an environmental stress factor was sufficient [134,135,136]. Deep sequencing of F1 sperm revealed upregulation of various miRNAs, which led to similar behavioral phenotypes in their offspring [136]. Thus, germ cells have extensive RNA patterns, and their complex profiles are increased by the chemical modification of RNAs, such as methylation [137], providing an additional layer of epigenetic information that might be transmitted to the next generation.

## 6. Evidence in Model Organisms: From One Generation to the Next Generations

There is growing evidence of the impact of adversity in early life or environmental stimulus on offspring where the contribution of epigenetic alterations in memory formation has been described [8,138,139,140]. One of the best-understood models in this field is the nematode *C. elegans*. Their features, such as its short lifecycle, the amenability for controlling many environmental and genetic variables, and the facility to continuously keep track of phenotypic changes during many generations, make them an advantageous experimental model for TEI research. Although the RNAi mechanism is the best studied in worms [141,142], robust TEI of both active and repressive histones PTMs were also described. In contrast, as we mentioned above, DNA methylation is not detectable in this model, as observed in *D. melanogaster* (Table 2).

Furthermore, several well-controlled research paradigms have been developed in rodents where an external stimulus leads to the phenotype changes in subsequent generations. Many of these works have focused on dietary intervention or behavioral studies. Outcomes of some studies on how epigenetic changes induced by different environmental experiences can contribute to subsequent generations’ cognitive function is summarized in Table 3.

## 7. The Relevant Contribution of TEI in AD Heritability

It is well established that age-related diseases are the result of the accumulation of epigenetic marks acquired throughout life, although at the same time, some works also reported that these modifications could have occurred during early adult life [156]. Nevertheless, what is more interesting, as we see throughout this review, the scientific evidence seems to describe that the transmission of acquired epigenetic traits is possible and is even maintained across multiple subsequent generations (TEI) [157], which might contribute to an increased risk of developing diseases, such as neurodegenerative diseases.

Taking into account that there are two types of AD: familial or early-onset AD (EOAD) (<1% of all AD cases), and sporadic or late-onset AD (LOAD) (>99% of cases). On the one hand, segregation analyses of EOAD cases have been linked to mutations in the genes encoding *APP*, presenilin 1 (*PSEN1*), and *PSEN2* [158], which are involved in the main molecular mechanism of AD pathogenesis, triggering the cascade of amyloid-β deposition, resulting in cognitive impairments. Nevertheless, these mutations explain only 5–10% of the occurrence of EOAD. On the other hand, the genetics of LOAD is much more complex than that of EOAD. Genome-wide association study (GWAS) has been carried out to elucidate the remaining genetic risk for AD, identifying over 20 genomic loci [159]. One of the most robust genetic risk loci for LOAD is the apolipoprotein E gene (APOE) ε4. However, those genetic changes only explain around a quarter of the total heritability, thereby AD seems to be poorly driven by genetics [160,161]. In fact, the value of APOE ε4 in predicting disease is limited since it is neither necessary nor sufficient to cause the disease. Up to 75% of individuals heterogeneous for APOE ε4 do not develop AD during life, and up to 50% of people with AD do not carry the high-risk ε4 allele [161]. Thus, most disease heritability remains unaccounted for, and the concept of ‘missing heritability’ has gained great attention [162].

Studies in monozygotic twins in humans provide the most accurate way to estimate the disease heritability. In 2006 a large twin study reported that the heritability for AD was estimated to be 58% in the full model and 79% in the best-fitting model, with the balance of variation explained by nonshared environmental influences [163]. The occurrence of phenotypical differences in monozygotic twins over time is thought to arise from epigenetic changes induced by different environments or stochastic events [164], thereby strengthening the idea that epigenetic changes resulting in altered gene expression may also be involved in the pathogenesis of LOAD. More interesting, LOAD’s etiology support that environmental factors through epigenetic phenomena are likely to contribute to the pathology progression, modifying disease risk and health outcomes [165,166,167]. Thus, this idea might help explain, in part, why some family members have a higher predisposition to certain diseases, as it depends on the environment–epigenome interactions that evolve during their individual life course.

In fact, a systematic review and meta-analysis based on the current evidence propose 21 nongenetic factors for the prevention of AD, such as diabetes, depression, hypertension, obesity in late life, depression, and stress, among others [168]. Accordingly, these effects of environmental stimuli during pregnancy, parental care, adulthood, and germline transmission have all been suggested as possible precursors of epigenetic changes that can be inherited transgenerationally [153,169]. How these nongenetic factors affect AD are not fully established. However, for instance, environmental enrichment (EE) contributions to cognitive improvement in 5xFAD and SAMP8 seem to support those epigenetic marks are important players [170,171]. Moreover, it is well established that during the prenatal and postnatal periods of brain development [172,173], there is an enhanced sensitivity to environmental factors, for example, key mediators of neural plasticity, such as BDNF, nerve growth factor (NGF), NT3 protein levels, expression of N-methyl-D-aspartate receptor subunits, and measures of long-term synaptic potentiation are strongly affected [174,175,176]. For example, long-term EE promoted neural plasticity through increasing levels of growth factors such as BDNF and NGF [177] via changes in DNA methylation of gene promoter in rat hippocampus [178].

Notwithstanding, both in humans and animals, there is little evidence about the molecular and epigenetic mechanisms underlying these heritability processes across generations in neurodegenerative diseases, including AD (Table 4).

## 8. Conclusions and Future Perspectives

It is well established that genetics only explain a small part of the total heritability, leading to different environmental stimuli and lifestyles that are important in the predisposition of human diseases. Experiments in model organisms have demonstrated that acquired traits from environmental or lifestyle factors may be responsible for the influence of genetic variability in subsequent generations. As we described above, we outlined the epigenetic contribution in the CNS’s regulation, modulating cognition function, and learning and memory formation. Moreover, several studies showed that impairment of epigenetic mechanisms promotes the alteration of gene expression underlying several age-related diseases. Hence, neurodegenerative disorders result from alterations in multiple molecular pathways together with the interaction of environmental factors, such as AD. The prevalence of AD is increasing during the last year, and although great efforts are focused on understanding the mechanisms involved in the pathology, none of the approved treatments turned out to be a total success. Hence, new insights in this field are urgently necessary. AD is considered multifactorial diseases due to the complexity of its aetiology, which appears to have at least a partial epigenetic aetiology. In fact, the LOAD represents 99%, and the genetic load only explain a small part of the cases. Twin studies strengthen environmental factors through epigenetic phenomena are likely to contribute to the pathology progression.

Given that epigenetics is the bridge between the environment and the epigenome, epigenetic alterations could contribute to improve our understanding of disease risk and health outcomes. Their potential reversibility allows predicting future disease risk and validating new therapeutic targets, as epigenetic intervention can modify the hippocampal transcriptome, potentially reversing age-related cognitive dysfunction. Epigenetics, therefore, is of considerable translational importance in the field of neuroprotection.

In fact, epigenetic alterations of DNA, histones, and ncRNAs, and subcellular or related structures can be inherited through the germline causing important changes in the phenotype of the offspring. This review has outlined some of the evidence for an epigenetic inheritance, both intergenerational and transgenerational, providing examples of how such regulation can contribute to the process of learning and memory. An example of the TEI phenomena described that temperature-induced change in expression from a transgenic *C. elegans* was maintained for up to at least 14 generations through histone methylation. Likewise, RNAi-induced TEI involved the heritable silencing by *set-25*. These findings point out to *set-25*, an ortholog of human G9a methyltransferase, as an important target to explain this phenomenon. On the other hand, maternal RSV supplementation prevented cognitive impairment in mice offspring, as has also been observed after RSV-supplemented HFD, by DNA methylation. BDNF DNA methylation was altered by prenatal stress induction, and thus proposing DNMTs as important targets.

In summary, these studies demonstrate that alterations in epigenetic modifications and their regulatory enzymes are capable of being acquired by offspring, orchestrating pathways related to cognitive function. Further studies will help shed light on these processes, pointing out new targets as a source of potential biomarkers diagnosing neurodegenerative diseases and as a potential target for therapeutic strategy.

## Figures and Tables

**Table 2:** Evidence of EI phenomena in nematodes and flies.

Model	M or P Inh	Experimental Design	Mechanism Lo-of-Function	Epigenetic Alteration	Up to	Outcomes	Refs
*C. elegans*	M,P	Learned behavior avoidance of pathogenic bacteria	Piwi/PRG-1		F4	TEI of *Pseudomonas aeruginosa* avoidance.	[143]
	M,P	Gene silencing	*set-25* and *set-32* mutation	H3K9me3	F3	RNAi-induced TEI involves initiation of silencing by canonical RNAi pathway genes, establishing heritable silencing by *set-25* and *set-32*, and ongoing maintenance of heritable silencing requiring small RNA-associated genes such as *hrde-1* and *nrde-2*.	[144]
	M,P	Temperature-sensitive transcriptional repression during 5 generations	*set-25* mutation	H3K9me2/3	F14	Reactivation of SET-25-silenced transposons. Inheritance occurs through both oocytes and sperm.	[145]
	M,P	Gene silencing	*Hrde-1/Wago-9*, and, *set-25* and *set-32*	piRNAs	F24	Germline nuclear small RNA/chromatin pathway can maintain stable inheritance for many generations when triggered by a piRNA-dependent foreign RNA response.	[132]
	M,P	Epigenetic memory	*mes-4* mutation	H3K36me	F1	MES-4 transmits the memory of gene expression in the parental germline to offspring, and that this memory role is critical for the PGCs to execute a proper germline program.	[146]
	M,P	Epigenetic memory	*Spr-5* (KDM1) mutation	H3K4me2	F30	The progressive derepression of genes that regulate spermatogenesis, defects in oogenesis and spermatogenesis and sterility	[147]
*D. melanogaster*	M,P	Heterochromatin organization	High-temperature induced p-Atf-2	H3K9me2	F5	Reduction of H3K9me2, disruption of heterochromatin formation and gene silencing	[148]

Abbreviations: M: maternal; P: paternal; inh: inheritance; PGR-1: Piwi (fruitfly) related gene; *Hrde-1*: heritable RNAi defective 1; *Nrde-2*: nuclear RNAi defective-2; p-Atf-2: phosphorylated activating transcription factor 2; piRNA: Piwi-interacting RNA; *Spr-5*: suppressor of presenilin defect 5; KDM1: lysine (K)-specific demethylase 1.

**Table 3:** Contribution of EI in the learning and memory process in rodent models.

Model	M or P Inh	Experimental Design	Mechanism Loss-of-Function	Epigenetic Alteration	Up to	Outcomes	Refs
Balb/C mice	M	Intergenerational transmission of aversive exposure attenuates Cognitive and Molecular	E-Cigarette exposure	DNA methylation	F1	Significant changes in global DNA methylation associated with significant changes in chromatin modification enzymes in the brains of the offspring. Maternal exposure to e-cigarette aerosols resulted in both cognitive and epigenetic changes in offspring were found.	[149]
CRND8 mice	M	Exercise during pregnancy	Early-life exposure	DNA methylation	F1	Exercise during pregnancy provides long-lasting protection from neurodegeneration and improves brain plasticity in the otherwise unstimulated progeny.	[150]
Wistar rats	M,P	Epigenetic memory	Early-life exposure to permethrin	5-mC 5-hmC	F1	Since the F1 generation did not receive any permethrin, the impairments observed in DNA methylation and hydroxymethylation, together with a reduction in dopamine levels in the F1 generation, have to be associated with parental early-life exposure to permethrin.	[151]
	M	Epigenetic reprogramming	Early life or prenatal stress induces	DNA methylation	F4	HSS decreased learning and memory of adult offspring in BPS and PS1, prominently.	[152]
Sprague-Dawley rats	M	Intergenerational transmission of alcohol consumption	Early exposure to alcohol	DNMT1 DNMT3a HDAC2	F1	Alcohol around the time of conception leads to sex and age specific behavioral adaptations later in life, along with gene expression changes to the methyltransferases, histone modifiers and other genes important for learning and memory.	[153]
	P	Epigenetic reprogramming	Exposure to cocaine	H3K4me1 H3ac	F1	Epigenetic changes in the hippocampus of male progeny associated with open chromatin states were found.	[154]
Long-Evans rats	M	Epigenetic reprogramming	Early life or prenatal stress induces	DNA methylation	F1	Early maltreatment produced persisting changes in methylation of *BDNF* DNA that caused altered *BDNF* gene expression in the adult prefrontal cortex. Altered *BDNF* DNA methylation in offspring of females that had previously experienced the maltreatment regimen.	[140,155]

Abbreviations: ac: acetylation.

**Table 4:** Examples of EI in AD mice models.

Model	M or P Inh	Experimental Design	Mechanism Loss-of-Function	Epigenetic Alteration	Up To	Outcomes	Refs
SAMP8 mice	M	Intergenerational transmission of diet attenuates Cognitive and Molecular	HFD	5-mC *Dnmt1* *Dnmt3a* m^6^A	F2	A significant increase in DNA methylation levels. Significant increase of m^6^A levels in HFD+RSV F1 and changes in gene expression of its enzymes *Mettl3* and *Fto*.	[179]
	M	Intergenerational transmission of diet attenuates Cognitive and Molecular	Supplementary diet	5-mC/5-hmC *Dnmt3A/B* *Tet2*	F2	Maternal resveratrol supplementation could prevent cognitive impairment in the SAMP8 mice offspring through epigenetic changes and cell signaling pathways.	[180]
CRND8 mice	M	Exercise during pregnancy	Early-life exposure	DNA methylation	F1	Exercise during pregnancy provides long-lasting protection from neurodegeneration and improves brain plasticity in the otherwise unstimulated progeny.	[147]

Abbreviations: m^6^A: N^6^-methyladenosine; HFD: high fat diet; RSV: resveratrol; Mettl3: methyltransferase like 3; Fto: FTO *α*-ketoglutarate dependent dioxygenase.

## Data Availability

Not applicable.
